# Development of the mental health cultural adaptation and contextualization for implementation (mhCACI) procedure: a systematic framework to prepare evidence-based psychological interventions for scaling

**DOI:** 10.1017/gmh.2021.5

**Published:** 2021-02-19

**Authors:** Manaswi Sangraula, Brandon A. Kohrt, Renasha Ghimire, Pragya Shrestha, Nagendra P. Luitel, Edith van’t Hof, Katie Dawson, Mark J. D. Jordans

**Affiliations:** 1 Transcultural Psychosocial Organization Nepal, Baluwatar, Kathmandu, Nepal; 2 Department of Psychiatry and Behavioral Sciences, George Washington University, Washington, DC, USA; 3 Department of Mental Health and Substance Use, World Health Organization, Geneva, Switzerland; 4 University of New South Wales, Sydney, Australia; 5 Centre for Global Mental Health, Institute of Psychiatry, Psychology, and Neuroscience, King's College London, London, UK

**Keywords:** Cultural adaptation, developing countries, group interventions, humanitarian crises, mental health, psychological distress, task-sharing

## Abstract

**Background:**

Because of the high burden of untreated mental illness in humanitarian settings and low- and middle-income countries, scaling-up effective psychological interventions require a cultural adaptation process that is feasible and acceptable. Our adaptation process incorporates changes into both content and implementation strategies, with a focus on local understandings of distress and treatment mechanisms of action.

**Methods:**

Building upon the ecological validity model, we developed a 10-step process, the mental health Cultural Adaptation and Contextualization for Implementation (mhCACI) procedure, and piloted this approach in Nepal for Group Problem Management Plus (PM+), a task-sharing intervention, proven effective for adults with psychological distress in low-resource settings. Detailed documentation tools were used to ensure rigor and transparency during the adaptation process.

**Findings:**

The mhCACI is a 10-step process: (1) identify mechanisms of action, (2) conduct a literature desk review for the culture and context, (3) conduct a training-of-trainers, (4) translate intervention materials, (5) conduct an expert read-through of the materials, (6) qualitative assessment of intervention population and site, (7) conduct practice rounds, (8) conduct an adaptation workshop with experts and implementers, (9) pilot test the training, supervision, and implementation, and (10) review through process evaluation. For Group PM+, key adaptations were harmonizing the mechanisms of action with cultural models of ‘tension’; modification of recruitment procedures to assure fit; and development of a skills checklist.

**Conclusion:**

A 10-step mhCACI process could feasibly be implemented in a humanitarian setting to rapidly prepare a psychological intervention for widespread implementation.

## Background

Prevalence of psychological distress is high among populations in low- and middle-income countries (LMICs) that are affected by conflict, poverty, and violence (Thornicroft *et al*., 2017; Charlson *et al*., 2019). LMICs are often unable to cope with high rates of distress, due to fragmented health systems and limited number of mental health professionals (Jordans and Tol, 2013). In such contexts, interventions are needed that can be scaled-up to reach larger populations, can be delivered through routine health care, and utilize concepts of task-sharing, i.e. use of non-specialists to deliver intervention components (Patel *et al*., 2018). Along with expanding the reach of interventions, it is necessary to assure that programs are culturally compelling (Panter-Brick *et al*., 2006). Global mental health efforts have also previously been criticized for cultural insensitivity and lack of alignment with local norms, family structures, daily practices, and cultural conceptualizations of distress (Summerfield, 2013). Changing the treatment without modifying implementation strategies may lead to limited delivery or uptake of the new services (Jordans and Kohrt, 2020). Therefore, cultural adaptation of both content and implementation strategies are needed for effective scale-up (Chambers and Norton, 2016).

### Overview of cultural adaptation methods

The main intention for cultural adaptation frameworks is to increase the cultural acceptability and effectiveness of the psychological treatment. This is accomplished by making changes that align with the culture of the beneficiary population, while maintaining the components of the evidence-based research that supports the treatment (Bernal and Scharró-del-Río, 2001; Sue, 2003; Fisher *et al*., 2014). There have been many attempts to organize the theories suggested by the existing frameworks. We have condensed these theories into three main distinctions that need to be balanced when adapting treatments (Griner and Smith, 2006; Bernal and Domenech Rodriguez, 2012, p. 20; Huey *et al*., 2014; Hall *et al*., 2016; Nisar *et al*., 2020).

The first distinction is *surface v*. *deep* adaptations. *Surface structure* adaptations refer to modifying superficial characteristics, such as translating treatment materials, to better fit preferences of the beneficiary population (Burge *et al*., 1997; Ahluwalia *et al*., 1999). *Deep structure* adaptations target cultural values, norms, traditions, beliefs, and the beneficiary population's perceptions of the illness's treatment and etiology. Surface level adaptations, such as pictorial material depicting participants ethnically similar to beneficiary populations, resulted in discernable improvements in participant retention (Harachi *et al*., 1997; Kumpfer *et al*., 2002) highlighting the importance of even ‘simple’ cultural adaptations (Chowdhary *et al*., 2014).

The second distinction is between the adaptations of *core v*. *peripheral* aspects of the intervention. *Core* components are the main evidence-based ingredients of an intervention that are integral to the treatment (Chu and Leino, 2017; Nisar *et al*., 2020). *Peripheral* components are related to the acceptability and feasibility of the intervention and exist to support the core components and the goals of the treatment. Although promoting adaptations that are responsive to the needs of the beneficiary population, it is also important to follow the intervention as intended. This *fidelity v*. *fit* distinction must be balanced to promote cultural appeal to the intervention while also following tried and tested methods to increase effectiveness (Castro *et al*., 2004; Kilbourne *et al*., 2007). Despite the clarity brought about by identifying three key distinctions in the literature, these aspects of cultural adaptations are not straightforward. For example, common factors in psychotherapy such as therapeutic alliance, changing expectations of personal effectiveness, and encouragement (Cuijpers *et al*., 2019; Heim and Kohrt, 2019) could be classified as *peripheral*, or supportive components, but in fact may be *core* and integral to the success of the treatment.

Despite a range of theoretical lenses to view cultural adaptation, the adaptation procedures have been criticized as difficult to replicate in real-world settings and lacking in transparency (Chu and Leino, 2017; Escoffery *et al*., 2018). Additionally, most adaptation studies of psychological treatments have been conducted with ethnic minorities in high-income countries (HICs) (Bernal *et al*., 2009). Although this is an overlooked and often marginalized population, these minorities are based in settings where resources such as time and personnel may not be as constrained as in low-resource and humanitarian settings that require rapid implementation. These constraints create unique needs, especially in LMICs and humanitarian settings, where a transparent, thorough, and prescriptive framework is necessary to guide rapid and systematic adaptions.

### Aim

Our aim was to develop a cultural adaptation model that addresses gaps in current approaches. We piloted our adaptation approach in the context of Group Problem Management (PM+) in Nepal (Sangraula *et al*., 2020). Group PM+ is a five-session intervention delivered by lay health workers, for adults with symptoms of common mental health problems (e.g. depression, anxiety, stress, or grief) (Dawson *et al*., 2015). The therapeutic aim of the intervention is to improve one's management of practical problems (e.g. work burden and relationship conflict), by utilizing four core strategies, or mechanisms of action (see Table 1). Participants are encouraged to practice and review these mechanisms of action in between sessions. Each session is 90 min and each PM+ group has 6–8 participants.

**Table 1.** Mechanisms of action of intervention

Intervention mechanisms of action	Description of mechanism	Implementation of mechanism
Stress management	Participants learn deep breathing. They are encouraged to incorporate this mechanism into daily life (e.g. when doing housework, walking). Grounding techniques are incorporated to bring participants back to the present.	Sessions 1–5
Managing problems	Participants learn which of their problems are solvable and which are unsolvable. One solvable problem is chosen and participants brainstorm solutions, then identify manageable steps to implement their solutions and accomplish their goals.	Sessions 2–5
Behavioral activation	Participants review the inactivity cycle. They choose a small activity that they enjoy doing (e.g. making and drinking tea, meeting a friend) or a task they need to complete and create a detailed plan about when and how to conduct this activity as a first step in breaking the inactivity cycle.	Sessions 3–5
Strengthening social support	Participants learn to recognize who among their family and friends are existing and potential sources of support and how best to strengthen connections with them. Participants could also identify broader community and organizational forms of support. Social network mapping activities are incorporated in this mechanism.	Sessions 4–5

*Note*: The first four sessions of PM+ each address a specific mechanism of action. The fifth and last session is a review of the mechanisms of actions learned in the intervention.

When conducting the cultural adaptation process for Group PM+, we documented the process and identified key adaptations. After implementation, we gathered data on how the adapted and contextualized intervention was perceived by participants, community members, and families. This paper aims to meet the following objectives:

1. Create explicit guidance for cultural adaptation and contextualization that can be applied to various populations with a focus on mechanisms of action, implementation, scalability, and quality monitoring.
2. Report on the cultural adaptation process of Group PM + in Nepal.
3. Gather feedback from program stakeholders to evaluate the implementation of the adaptations.
4. Suggest the most valuable steps in adapting an intervention under time and resource constraints.

The resulting methodology is the mental health Cultural Adaptation and Contextualization for Implementation (mhCACI) procedure. This paper will first outline the step-by-step mhCACI adaptation procedure followed by an overview of the key adaptations made to the Group PM+ intervention. We will then highlight how this procedure can be used to adapt future interventions and treatments in various contexts.

Although an existing cultural adaptation methodology has been presented to adapt individual PM+ with refugee populations (Perera *et al*., 2020), our approach has several advantages including a central focus on the processes that lead to change (i.e. mechanisms of action), attention to implementation parameters beyond content modification (e.g. how do participants become engaged in the intervention and how is it explained to the public and families), rigorous documentation, and creation of a cultural distress model that goes beyond simply translating mental health terminology.

## Methods

### Setting

Nepal is a low-income country with a history of internal conflict, political instability, and natural disasters. In 2015, an earthquake resulted in injuries, deaths, and displacement. A total of 34.3% and 33.8% of participants in an earthquake-affected district scored above the validated cut-off scores for depression and anxiety, respectively (Kane *et al*., 2018). To date, there have been minimal efforts to adapt psychological treatments in Nepal (Ramaiya *et al*., 2017). Group PM+ was considered an appropriate intervention to test in this setting due to its scalability and task-sharing approach. The Group PM+ intervention's adaptation process and feasibility trial was conducted in Sindhuli district, which was heavily impacted by the 2015 earthquake (Sangraula *et al*., 2018, 2020). The adaptation process and implementation of the trial was conducted by the staff of Transcultural Psychosocial Organization (TPO) Nepal based in Kathmandu, Nepal (Upadhaya *et al*., 2014).

### Ecological validity model (EVM) and replicating effective programs (REP) frameworks

We built the mhCACI procedure upon the Ecological Validity Model (EVM) to guide our adaptation of the Group PM+ intervention content (Bernal *et al*., 1995; Bernal and Sáez-Santiago, 2006). The EVM was selected because it is based on the view that individuals must be understood within their cultural, social, and political environment. The EVM framework serves to ‘culturally center’ an intervention through eight dimensions that must be incorporated for an intervention to have ecological validity and be embedded within the cultural context (Bernal, 2003). These dimensions include language, persons, metaphors, content, concepts, goals, methods, and context (Table 1 in online Supplementary material).

We also structured the adaptation and contextualization methodology within the Replicating Effective Programs (REP) framework, which considers intervention training, supervision, and fidelity assessments as crucial components to the implementation of effective interventions while allowing for local flexibility (Kilbourne *et al*., 2007). REP includes four phases: (1) pre-conditions (e.g. identifying need and target population), (2) pre-implementation (e.g. community input and training), (3) implementation (e.g. program delivery and evaluation), and (4) maintenance and evolution (e.g. re-customization and preparing intervention for implementation in another context or dissemination) (Cabassa and Baumann, 2013).

### Study methodology

This study was registered in ClinicalTrials.gov (NCT03359486). As part of the 10-step cultural adaptation model, qualitative interviews were conducted with program stakeholders to gather feedback on the acceptability, scalability, and implementation of the adaptations (Sangraula *et al*., 2020). Integrated within the maintenance and evolution phase, a subsequent Group PM+ evaluation will be conducted in Nepal with the integrated changes in implementation methods (van't Hof *et al*., 2020).

### Overview of mental health cultural adaptation and contextualization for implementation (mhCACI) methodology

The adaptation process was an ongoing, iterative process with some overlapping steps. Questions addressed in each step were based on what was or was not answered in the prior steps and the iterative process of this methodology more easily allowed for finding a balance between *fidelity v. fit*. The format of this methodology was participatory and involved a high level of engagement with the communities where the intervention was delivered.

A detailed data collection process and documentation system allowed us to ensure that each adaptation made was based on evidence. We created a matrix before the start of the adaptation process based on the FRAME approach for documentation (Wiltsey Stirman *et al*., 2019): (1) the eight broad dimensions from EVM, (2) implementation strategy (what exactly should be changed in the intervention material), (3) rationale for change (description of why it should be changed and what it would accomplish for the intervention), and (4) evidence for change (which adaptation steps the change was a result of). All changes and adaptations were listed in the EVM matrix during the length of the process (Table 3 in online Supplementary material).

The following approach charts the cultural adaptation process from preparation through implementation (see Table 2). We present the steps as they would fit into the first three phases of the REP framework (pre-conditions, pre-implementation, and implementation).

**Table 2.** Overview of adaptation steps: activities, participants, and methods of analysis outlined according to phases of the Replicating Effective Programs framework

Adaptation step	Objectives	Activities	Participants	Data collection and methods of analysis	Duration
**Phase I: Pre-condition**					
*Identify need for intervention, identify intervention for local setting, package intervention for training and assessment*					
(1) Identifying mechanisms of action	To identify the main ingredients of the intervention that lead to outcomes and cannot be drastically modified	Collaborate with researchers/personnel that developed the intervention Conduct background reading and research on the core techniques and activities of the intervention	Researchers (4), Clinical supervisors (2)	Summarize each mechanism of action and solidify the team's understanding of these core concepts	1 week
(2) In-depth literature review	To identify issues for engagement/implementation related to mental health research and services in program site, To gather information on previously conducted programs/research for site and population of interest	Conduct a systematic review of existing literature Identify experts in the field of service delivery and interview	Coders (2), Articles (43), Interviewers (2), Experts interviewed (7)	Screen articles for those that address relevant interventions in program site, Extract data from selected articles on delivery agents, trainings, supervision, process, and outcome measures, psychoeducation methods, integration into health system, and cultural/ethnopsychology elements, Summarize data for key findings, recommendations for intervention and gaps in research and methodology	2 months
**Phase II: Pre-implementation**					
*Orientation to core elements, customize delivery, identification of barriers, staff training needs, technical assistance needs*					
(3) Training of trainers (ToT)	To incorporate pre-existing practices in program site, To identify how existing training transmits mechanisms of action, To identify overlap of approach with preexisting practices in program site	Training of counselors and clinical supervisors on delivering intervention trainings to delivery agents (CPSWs)	Expert clinical supervisor (from a previously conducted intervention site) (1), Local clinical supervisors (2), Additional counselors for intervention support (6)	Participants of ToT write suggested adaptations directly in the manual, Clinical Supervisors review suggested adaptations after the ToT before finalizing into the manual	10 days
(4) Translation of the manual	To translate the intervention manual (and additional intervention material) from English to program site language	Translate manual (and additional intervention material) from English to program site language	Translator (1), Clinical supervisors (2)	Clinical supervisors conduct frequent meetings with the translator to verify that the language is easy to understand for delivery agents	3 months
(5) Expert read through	To gain additional perspective on context, content, language, and applicability from persons experienced in the program site	Expert counselors read through manual and program material and suggest changes	Counselors (3), Clinical supervisors (2)	During a 1-day workshop, counselors read through the manual together and note necessary changes based on the eight dimensions of the Bernal framework	1 day
(6) Formative qualitative study	To gather information on acceptability and applicability of the intervention in program site, To identify existing resources in the community as sources of referral	Conduct KIIs and FGDs with community members and key stakeholders in study site, Focus questions on understanding community's awareness of mental health, identification of pre-existing mental health and other resources in the community, identify practical problems to adapt in the manual	Local stakeholders, FCHVs, local health workers (18+), Interviewers (3)	Create interview guide to address remaining questions on resources in the community, level of awareness on topic of interest, and program implementation details, Conduct KIIs and FGDs with a variety of local stakeholders to gather varying perspectives (e.g. for Group PM+, 18 KIIs and 2 FGDs were conducted with local community members, mother's group members, FCHVs, and other key informants), Code interviews using deductive analysis and summarize key findings related to program implementation and cultural elements for the manual, Apply findings from qualitative analysis to the manual, other program material, and program implementation strategy	1.5 months
**Phase III: Implementation**					
*Ongoing community partnership, booster trainings and supervision, process evaluation, and feedback and refinement of intervention package and training*					
(7) Practice rounds	To provide first-hand experience to program site supervisors in delivering the intervention and to make further changes to intervention material from this experience	Clinical supervisors conduct all sessions of the intervention with target populations (e.g. for Group PM+, all 5 sessions were conducted with one female and one male group)	Clinical supervisors (2), Female participants (6), Male participants (6)	Facilitators (clinical supervisors) of intervention noted if adaptations already in the manual were feasible and acceptable among practice round informal participants	2–3 months
(8) Team adaptation workshop	To summarize all intervention adaptations before implementation	Discuss all suggested adaptations, Finalize adaptations thus far and document accepted adaptations in an EVM overview	Program staff (principal investigators, program coordinator, clinical supervisors) (6)	Prepare the EVM matrix (see online Supplementary material) to summarize adaptation principles, page needed to be changed within manual, implementation (what should be changed), rationale (why it should be changed), and evidence (which adaptation method informed suggested change), Finalize adaptations thus far by adding all small or large changes in EVM matrix (online Supplementary material)	1 day
(9) Implementation and supervision	To gather feedback from the implementation and supervision process to further adapt intervention, To summarize experiences using the intervention manual and proposed intervention implementation strategies and scalability	Conduct intervention facilitator training, Gather any suggestions for changes from the facilitator training, Conduct intervention, supervision and implement all program activities, Record detailed notes about first-hand experiences on implementing intervention	Clinical supervisors (2), Delivery agents (8), Research supervisor (1), Program coordinator (1), Program participants (60)	Trainers (clinical supervisors) record suggested adaptations, during training of delivery agents, directly into the manual and review before finalizing for implementation within the program, Field and program staff record process notes reflecting daily on experiences using the manual, feasibility of the intervention, community perceptions, supervision, challenges, and suggestions for change	6 months
(10) Review through process evaluation	To gain perspective on the successes and challenges of varying adaptations and implementation strategies, as experienced by community stakeholders and program participants, to address during the definitive trial, To gather information from field staff on adaptation suggestions and improvements to increase the feasibility, acceptability, and fidelity of the program	Conduct KIIs and FGDs with community members, key stakeholders and local staff in program site, Focus questions for trial participants, family members, local health workers, and community leaders on feasibility, acceptability and implementation of intervention, Focus questions for staff on intervention and program fidelity, and challenges in feasibility and acceptability	Intervention facilitators (8), Research assistants (8), Intervention participants (8), Intervention participants' families (3)	Synthesize data through FGDs with field staff and in a workshop with program team to discuss changes necessary for the definitive trial, Conduct KIIs and FGDs with a variety of local stakeholders to gather varying perspectives (e.g. for Group PM+, 31 KIIs and 6 FGDs were conducted with local community members, FCHVs, mother's group and other key informants), Code interviews using deductive analysis and summarize key findings related to main dimensions [Table 5] Apply findings from process evaluation to the manual and program implementation strategy for the future program implementation [Table 5]	2 months

### Phase I: pre-conditions

1. Identify the key mechanisms of action

The mechanisms of action are the process by which a psychological intervention reduces distress and supports behavioral change (Kazdin, 2007). Mechanisms of action, or the techniques participants learn in each PM+ session, were identified as the core of the intervention in previous trials (Dawson *et al*., 2015; Bryant *et al*., 2017; Rahman *et al*., 2019). Therefore, these techniques were also identified as the key mechanisms of action in this trial (see Table 1).

1. In-depth literature review (and consulting with experts)

A systematic review of existing literature on mental health interventions in Nepal was conducted. Databases such as PubMed, PsychInfo, and PsychiatryOnline were searched as well as gray literature from policy briefs and annual reports of local organizations. The review extracted information on cultural concepts of distress, Nepali ethnopsychological frameworks, and prior adaptations of mental health interventions in Nepal. Ethnopsychological frameworks are local models of understanding suffering, mind-body relationships, and concepts of the self (Kohrt *et al*., 2012). Although not a formal part of the literature review, interviews were also conducted with staff from leading mental health organizations in Nepal to identify issues for community engagement and implementation related to mental health research and service delivery in Nepal. Data were summarized for key findings, gaps in research and methodology, and recommendations for Group PM+ adaptation in Nepal.

### Phase II: pre-implementation

1. Training of trainers (ToT)

Clinical supervisors and supporting counselors were given a 10-day training by a Group PM+ trainer from a previous study site. The participants of this ToT identified overlap of approach with preexisting practices in the program site and suggested culturally fit adaptations to intervention content. The Group PM+ clinical supervisors gathered the adaptations suggested by the trainees and reviewed them before finalization. Suggestions that modified the mechanisms of action were rejected. Other suggestions that adapted other aspects of the intervention, such as the language or metaphors, were documented, accepted and finalized into the manual by the clinical supervisors.

1. Translation of manual

Clinical supervisors incorporated initial changes into the English manual which was then translated to Nepali by a professional translator. Clinical supervisors regularly reviewed the translator's progress to ensure that the manual could be understood by non-specialist delivery agents. This was an ongoing process and focused on language rather than the content of the manual. Study staff without a clinical background also reviewed the manual to ensure its comprehensibility for lay persons.

1. Expert read-through

Experienced bilingual Nepali psychosocial counselors read through the Nepali language intervention manual and suggested changes in language and content to fit into the cultural context during a 1-day workshop. The main objective of this step was to gain additional perspective from persons experienced in the program's mental health context on the intervention's content, language, and applicability.

1. Formative qualitative study

Based on gaps identified in prior steps, a formative qualitative study was conducted regarding community's awareness of mental health, collaboration with pre-existing community resources and identification of practical problems faced by community members. Key informant interviews (KIIs) (*n* = 18) and focus group discussions (FGDs) (*n* = 2) were conducted with female community health volunteers (FCHVs), leaders, health workers, and community members. Interviews were coded by two coders (MS and RG) using deductive analysis. Key findings related to program implementation and cultural elements were added to the manual and the program implementation strategy. This included the addition of community sensitization events and meetings with the families of participants, refining recruitment of participants, and clarifying referral pathways.

### Phase III: implementation

1. Practice rounds

Clinical supervisors conducted Group PM+ practice rounds to gain experience delivering the five-session intervention, gather feedback from the participants on their comprehension and relatability of the intervention, and apply any further changes to the manual and implementation strategy. Practice rounds were conducted with one male group and one female group from a nearby community. After each session, the participants were encouraged to give feedback to the facilitators on content, language, materials and methods used, and facilitation skills. This information was collected through informal interviews with the participants and noted down by the clinical supervisors.

1. Team adaptation workshop

A team workshop with all core program and research staff was conducted to summarize all intervention adaptations listed to date on the EVM matrix. Program staff also modified competency and quality monitoring procedures. Once all adaptations were thoroughly discussed, clinical supervisors made final changes to the manual before the start of the trial.

1. Program implementation, supervision, and process evaluation

Lay Nepali community members were recruited to deliver Group PM+ to their communities (Sangraula *et al*., 2018, p. 201, 2020). During the Group PM+ training, the facilitators were encouraged to suggest changes in the manual's language and feasibility and acceptability of the proposed implementation strategy. We employed a randomized control trial design where the two chosen Village Development Committee in Sindhuli district 120 participants were randomly assigned to enhanced usual care or PM+. As part of supervision, all staff recorded notes about first-hand experiences working on program recruitment, delivery, and engagement with the community, and shared these experiences with their supervisors. Some changes were made in real-time while others required further discussion at the end of the trial.

After completing the intervention, 31 KIIs and six FGDs were conducted with field staff, intervention participants, participants' families, and other key community stakeholders to gain perspective on the successes and challenges of varying adaptation and implementation strategies. A deductive data analysis process was used; key themes were identified prior to analysis and a codebook was developed (Sangraula *et al*., 2020). Interviews were coded using NVivo software and the two coders (MS and RG) established an acceptable inter-reliability rate (=0.8) during the coding process. Key implementation challenges, successes, and further suggestions for the program were extracted and summarized from the interviews.

1. Re-customization of intervention

Field notes from program implementation and supervision, along with results of the process evaluation were gathered and synthesized into the EVM matrix (Table 2 in online Supplementary material) to highlight suggested key changes for re-customization of the intervention. The program staff then discussed appropriate changes related to program content, recruitment methods, quality assurance, and strengthening the mechanisms of action. Program materials were further revised to reflect the outcomes of implementation assessment before implementation in a new context.

## Results

### Conceptualization of stress and tension

**Fig. 1.**
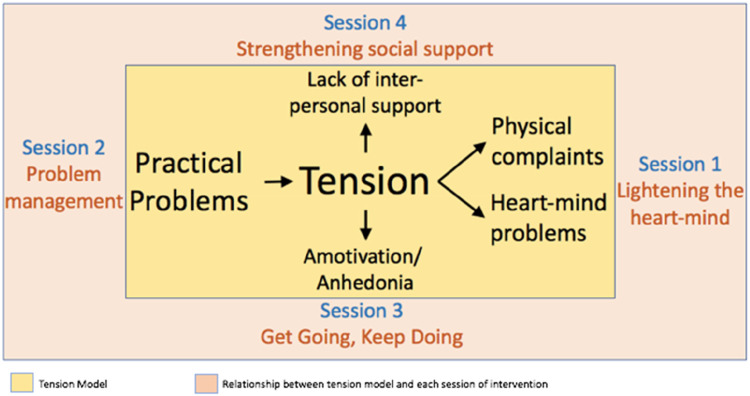
*Tension* conceptual model.

As a central focus of the adaptation process, we aimed to create a conceptual model by linking the mechanism of action to how distress was experienced in this context. *Tension* was used as a non-stigmatizing idiom of distress, as a proxy to depression complaints which is targeted by the intervention, and was commonly used in lay-Nepali language by community members of all ages, gender, and socioeconomic status (Rai *et al*., 2017). The *tension* ethnopsychology model was conceptualized during the workshop as the team was finalizing the adaptations before the trial (see Fig. 1).

According to the ethnopsychology model, adversity or practical problems lead to *tension*, which can have a physical manifestation and lead to emotional problems. *Tension* can also lead to a lack of energy, feeling unmotivated, and isolation from friends and family. Each Group PM+ session addresses managing the roots of *tension* or its effects, which are also integrally linked with one another. Because of the contextual fit of the model, elements of the *tension* model were also used during facilitator training and the recruitment process to explain the effects of adversity on our lives to local community members.

### Key adaptations

Adaptations were systematically documented in the EVM matrix (see online Supplementary material). Key adaptations are summarized in Table 3.

**Table 3.** Key adaptations from each step outlined according to phases of the Replicating Effective Programs framework

Adaptation steps	Key adaptations
*Phase I: Pre-conditions*	
(1) Identify mechanisms of action	Four key mechanisms of action were identified; stress management, managing problems, behavioral activation, and strengthening social support^4,6^. Because these mechanisms of action cannot be changed, adaptations in the next steps will be made to support the beneficiary population's comprehension of these techniques.
(2) In-depth literature review	The literature review suggested that mental illness is deeply stigmatized in Nepal and idioms of distress such as *man ko samasya* or *tension* can be used in rural communities to refer more openly to general distress. This suggests a need for developing a non-stigmatizing conceptual framework for this intervention to be used in the Nepal context^1,3,4,5,8^. Task-sharing trainings are common in Nepal, due to the lack of trained mental health professionals. Trainings are followed by frequent on-site and off-site supervision for the non-specialists. We decided that for Group PM+, Clinical Supervisors will conduct weekly office supervision and will observe two sessions per group^2,7^.
*Phase II: Pre-implementation*	
(3) Training of trainers (ToT)	Trainers suggested adding pre-existing counseling techniques to the Group PM+ intervention in order to strengthen the mechanisms of action. These techniques were previously proven to be effective in the Nepal context. Examples include grounding techniques (where participants are brought to the present moment by using senses to identify what is around them) and me-mapping (an activity where participants identify their close relationships in pictorial form)^4,5,7^. As part of the training, trainers read the manual and reviewed other implementation material thoroughly and suggested further language, such as *man ko samasya*, to decrease self-stigma. This aligned with the results of the literature review^1,5,7,8^. Trainers suggested adding multiple culturally appropriate ice breakers and energizers throughout the group sessions. This was to ensure that participants would stay on task throughout each session^4,5,7^.
(4) Translation of manual	Translation of the manual was an iterative process where the translator met often with study staff to review translations and to ensure that the language was simple and accessible for lay facilitators^1,4^. Although the translator was making progress on the manual, the Clinical Supervisors felt the need to create a fidelity checklist for the facilitators to follow during sessions and to use alongside the manual^7^.
(5) Expert read-through	Experts with experience counseling in Nepal stressed the importance of gender matching facilitators and participants for the intervention^2,7,8^. Family engagement was identified as a key component in prior mental health interventions in Nepal. A family meeting, to involve participants' families in the clinical process, was added as a component to Group PM+^2,4,5,6,7,8^.
(6) Formative qualitative study	Local community members identified that it is deemed acceptable for facilitators of lower caste to work with participants of higher caste in the local area. Therefore, facilitators from all castes can be hired to deliver the intervention^2,8^. Nearby resources such as health posts, mothers' groups, domestic violence NGOs, and safe homes were identified as referral services for intervention participants if needed^7,8^. Gender issues and social discrimination were identified as sources of stress for community members. It was suggested to add these issues in the manual and other clinical material z^4,8^.
*Phase III: Implementation*	
(7) Practice rounds	Participants in the practice rounds suggested adding more posters and visuals to the clinical content. They also suggested personal problems that characters may have for these materials. Examples included an unemployed man returning home from working a labor job abroad, a daughter-in-law having an argument with her mother-in-law, and a woman unable to concentrate on her work in the farm due to stress^3,4,8^. Clinical supervisors found that when running the practice sessions, participants would often arrive late and some would forget the day of the week the sessions were held on. This highlighted the importance of having a helper who could call the participants a day before the session as a reminder or gather them from their homes before the start of each session. Participants also suggested the distribution of a calendar during the first session as a reminder of when to attend the following sessions^4,7,8^. Clinical supervisors noted that some participants were dominant while others were quieter. It was suggested to increase training in group facilitation skills and managing dominant and quiet participants. Facilitators must also take special note of gender, socioeconomic status, ethnicity/caste and other social factors and their possible effects on group participation^2,4,7^. Some participants in the practice rounds expected monetary benefits from the intervention. Therefore, clinical supervisors found it necessary to clarify that the program is not for those who need support with their economic situation but is for those with *man ko samasya* and *tension*^4,8^.
(8) Team adaptation workshop	The role of the helper was further defined during the workshop. From experience gathered from each of the steps, the team agreed that the helper would also be responsible for administrative tasks, such as hanging posters and writing on the board, that would allow the facilitators to place all of their focus on the participants and session material^2^. Study staff found that it would be necessary to include a method to evaluate participants' progress and suicidal tendencies at the start of each session and to conduct referrals as necessary. It was decided during the workshop that the Psychological Outcomes Profile (PSYCHLOPS) would be administered during snack time at the start of each session^6,7^. A general distress version of the CIDT will be created and used for recruitment of participants^4,5,6,7,8^. The *tension* conceptual model was created during the workshop as program staff brought together various conceptual adaptations informed by the adaptation process. The conceptual model relates each session to an aspect of general distress^4,5^.
(9) Implementation and supervision	During the recruitment of program participants, the facilitators voted to change the Group PM+ program title to *Khulla Man*, meaning open-hearted, a more culturally appropriate and de-stigmatizing program name^3,4,8^. Community sensitization events were led by facilitators and program staff to raise awareness about mental health and recruit participants into the program. Over time, these events went from lecture heavy to discussions about the *man* (heart-mind) and the causes and symptoms of *man ko samasya*. This method of engagement was found to be especially helpful with recruitment and de-stigmatizing mental health issues^3,4,5,7,8^.
(10) Review through process evaluation	Facilitators mentioned that conducting practice groups supported them in feeling prepared before the start of the trial and recommended that the facilitators conduct more practice groups before the next trial^3^. Participants noted that although they enjoyed the sessions and practiced what they had learned at home, they sometimes had difficulty in remembering all the techniques learned. As part of the review after the pilot trial, a ‘tension toolkit’ was developed by study staff to help participants remember techniques learned in each session. The toolkit included cards for each session with pictures of the techniques learned, and a space to track how many times participants practiced each technique. A certificate of completion was also included to encourage participants to continue utilizing what they learned after the final session^3,4,6,7^.

Superscript numbers refer to domains of Bernal's Ecological Validity Model: ^1^Language, ^2^persons, ^3^metaphors, ^4^content, ^5^concepts, ^6^goals, ^7^methods, ^8^context.

### Pre-conditions

#### Identification of distress and relatability of clinical content

Understanding sources of distress was key to identifying need for intervention in the local community. Identified daily stressors, such as financial burden, grief, and migration, were incorporated into the stories used during sessions. Participants in the trial found these problems, the case story, and pictorial representations on posters to be highly relatable (Sangraula *et al*., 2020). Clinical supervisors and facilitators noted that physical health was a source of stress for the majority of program participants. The feedback received demonstrated that more content was necessary to address physical problems and train facilitators on how to work with participants who did not discuss their emotional problems. Some suggestions included adding characters in the case story that faced more physical ailments, and posters representing these problems to validate participants' distress. Facilitators should also be trained further in how somatic problems are connected to mental well-being, using the *tension* model as a guide.

### Pre-implementation

#### Use of local idioms to increase acceptability and reduce stigma

Community sensitization events were a vital component of recruitment to address the stigma and lack of awareness of mental health issues at the program site (Sangraula *et al*., 2020). Facilitators invited community members to discuss causes of *tension* and *man ko samasya,* which is Nepali term for heart-mind problems refering to general psychological distress (Kohrt and Harper, 2008), and impact of adversity on personal behaviors and emotions. The participatory format of engaging with the community during sensitization activities while using de-stigmatizing terms was successful in encouraging community members to volunteer for study screening. In further efforts to reduce stigmatization by the community for participating in the intervention and self-stigmatization by the participants themselves, key adaptations were made to the metaphors, the concepts, and the intervention packaging. A context appropriate name was voted on by the local staff that chose *Khulla Man*, (a Nepali idiom for opened heart-mind, meaning open-hearted). Some participants described their own heart-mind as being *Khulla* (open) or having a *Khulla Man* after the sessions. Similarly, the *tension* model conceptualized before program implementation was successful in supporting the facilitators' understanding of how each session aimed to reduce stress.

### Implementation

#### Recruitment, training, and supervision of intervention facilitators

Recruiting, training, and hiring local lay workers was a critical adaptation to increase scalability of the Group PM+ intervention. A 20-day Community Psychosocial Worker (CPSW) Training, as is standard to certify this cadre of workers in Nepal, was delivered before 10 days of Group PM+ training. As indicated by the qualitative interviews, participants found comfort in having their groups led by a facilitator who was from a familiar location but who the participants did not know very well. Similarly, helpers were hired to assist the facilitators during the sessions. Because concepts of time and punctuality were flexible in this context, it was noted that having a helper remind participants to arrive on time helped increase attendance and reduce drop-out rates.

### Implementation

#### Engagement with potential beneficiaries

The Community Informant Detection Tool (CIDT) helped easily identify members of the community experiencing mental health symptoms through using vignettes and pictures (Jordans *et al*., 2015). A general distress, *man ko samasya*, version was developed for this trial. FCHVs, mothers' group members, and other local community leaders received a 1-day training on how to use this tool to refer those with general distress to the study. They were also trained on the severe mental health CIDT version to identify who not to recruit. However, this led to confusion among some trainees who referred those with severe mental illness to the study, since severe symptoms are more noticeable than those with general distress. For future scalability and dissemination, it is recommended to train local community members on using the general distress version only and with regular on-site supervision.

### Reinforcement of mechanisms of action

A combined competency and fidelity checklist was created based on both Group PM+ elements and common factors in psychological treatments, drawn from the enhancing assessment of common therapeutic factors tool (Kohrt *et al*., 2015). Clinical supervisors attended at least two of the five sessions per PM+ group and used the fidelity checklist to measure facilitators' competency in delivering the intervention. Facilitators also used the checklist while conducting sessions to ensure that the mechanisms of action were addressed thoroughly. The Reducing Tension Checklist (RTC) tool was also developed to assess whether participants applied the mechanisms of action learned in the sessions to their daily lives. This tool was used pre- and post-intervention (Sangraula *et al*., 2020).

The participants noted that of the different techniques they learned, the deep breathing technique was the most memorable and used the most outside of the sessions (Sangraula *et al*., 2020). This technique was noted as the most tangible, accessible, and lead to the most immediate results. However, facilitators found it difficult to track how often participants were practicing at home.

As a result of the process evaluations, the team decided to incorporate more imagery and memorable metaphors to each of the sessions. For example, the second session focused on effective problem solving, was considered by the facilitators to be one of the most difficult sessions to deliver and for real-life application outside the sessions. Therefore, the definitive trial incorporated an image of a hand with a step for problem solving written on each finger. A card was created for each of the five sessions with visual imagery of each mechanism of action on one side and a space to plan when to practice on the other side. This set of cards was called the *tension* toolkit (Fig. 1 in online Supplementary material). We also incorporated several concrete tools to support each of the sessions, such as a small pouch (*thaili*) after the third session for participants to store a rock, or kernel of corn each time they do a pleasurable activity at home. The objective of these tools is to provide physical items to help participants practice skills.

## Discussion

### Outputs and applications

This study provides a methodology for the mhCACI that incorporates cultural adaptations to clinical content, scalability, and implementation. Because this cultural adaptation methodology has been integrated into the REP framework, this study further bridges the gap between cultural adaptation and implementation science research (Cabassa and Baumann, 2013; Mutamba *et al*., 2018). The scalability and implementation aspects were shown to be just as important as the clinical content when adapting an intervention. A *tension* ethnopsychology model was developed as a conceptual foundation to key adaptations. Adaptations were made to case stories, visuals, and materials to reflect the context. Using a context appropriate intervention name, utilizing the CIDT for recruitment, and conducting community sensitization events supported in reducing the stigma. We also adapted the facilitator trainings, and created the fidelity checklist, RTC, and tension toolkit to reinforce the mechanisms of action and ensure quality of care. As part of this study, we have also designed a clear and detailed documentation process that will assist in conducting evidence-based adaptations to future interventions.

The specific adaptations made to the intervention as well as the contextualization of the implementation process were demonstrated to be feasible and acceptable. The intervention had a high retention rate with 75% of the participants completed 4–5 sessions (Sangraula *et al*., 2020). All facilitators (*n* = 4) scored above a 75% on the fidelity checklist developed to measure the competency and overall fidelity to the intervention. The intervention group showed an improvement in outcomes, especially in general psychological distress. Qualitative interviews with the Group PM+ facilitators, supervisors, and beneficiaries suggest that benefits can be attributed to the cultural adaptations and contextualization process.

**Fig. 2.**
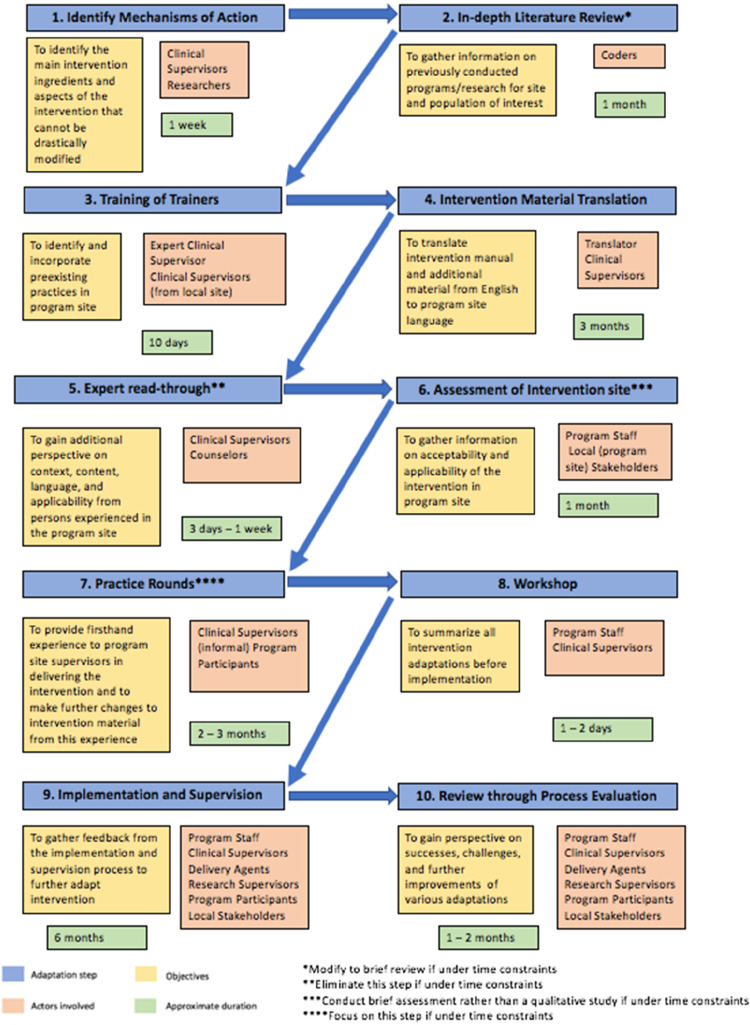
Cultural adaptation step-by-step guide.

In reality, adaptation processes are often conducted constrained by staff and time in low-resource settings. Therefore, we have created one mhCACI procedure with two versions: the first model to be used in contexts with at least modest time and resources, and the second in contexts with high-level constraints (see Fig. 2).

For the shorter mhCACI procedure, the literature review step has been modified from in-depth to an overall review. The expert read-through has been eliminated since we found that clinical staff who developed in-depth knowledge of the intervention when conducting prior adaptation steps were best fit to suggest the most meaningful changes. Though we conducted a formative qualitative study as part of the adaptation process, we found that an assessment of the intervention site could have sufficed for the purpose of adaptation. This assessment should be tailored to the needs of the intervention and should be approached as a method to gather information from and to engage the local community, which was proven to play a large role in the success of the intervention. The practice round steps resulted in the greatest number of adaptations and were important steps in addressing the logistical aspects of the intervention, such as time management, venue location, and to gather feedback from participants within the targeted population. Therefore, we recommend shortening other steps and focusing mainly on conducting several practice rounds or sessions as part of the adaptation process, if under extreme time constraints.

The eight dimensions of cultural adaptation, as presented by Bernal and colleagues (Bernal and Domenech Rodriguez, 2012), were helpful in conceptualizing the types of changes that could be made, while preserving the treatment's core mechanisms of actions. However, we found that a few of the dimensions, such as content and context, are similar in their definitions and overlapped with one another. As a result, we found it best to focus less on which category an adaptation would be labeled as, and instead refer to the eight dimensions occasionally to assure that they are all being addressed as a part of the adaptation. In this way, the eight dimensions can serve as a starting point rather than a guide.

### Limitations

Limitations to the study must be accounted for before using this adaptation model for other interventions. It is difficult to identify which adaptations were the most important and which had the highest impact on the intervention because there are many potential factors contributing to its success. The results are also limited to qualitative work and the participants', staff, and stakeholders' perceptions of effectiveness. Regardless of compressing the adaptation process to fit resource limited contexts, the nature of the cultural adaptation itself is iterative and requires depth and heavy documentation. If a single professional wishes to adapt a program and is especially limited on human resources, collaboration with external experts is recommended to complete the adaptation steps.

## Conclusion

This study proposes the mhCACI procedure as a clear and systematic adaptation process that can be conducted within implementation science methodologies, such as the REP framework. We provide documentation tools to guide the mhCACI procedure for future interventions. The combination of the REP framework and the 10-step methodology allows for a focus on intervention content, scalability, quality monitoring, and flexibility while maintaining fidelity. Although this process was used to adapt a mental health intervention in an LMIC setting, it can be used to adapt interventions for various populations, such as ethnic minorities in HICs or humanitarian settings. With the increase in interventions that employ the concept of task-sharing (Patel, 2012), this process also serves as an example for future interventions in LMIC settings. Although this process was used to adapt a group intervention, it is flexible enough to be used to adapt an individual intervention or even a treatment beyond mental health.

## Supplementary material

For supplementary material accompanying this paper visit http://dx.doi.org/10.1017/gmh.2021.5. click here to view supplementary material

## Data

Contact the authors for data request.
