# Supplementary material for: Development of the mental health cultural adaptation and contextualization for implementation (mhCACI) procedure: a systematic framework to prepare evidence-based psychological interventions for scaling
Source: Glob Ment Health (Camb). 2021 Feb 19;8:e6. doi: 10.1017/gmh.2021.5 (PMC8082944; doi:10.1017/gmh.2021.5)
Supplement: Supplementary file 1 [file S2054425121000054sup001.docx]

**Supplementary Material**

**TABLE 1.** Ecological Validity Model (EVM) Framework Dimensions

| **Dimensions** | **Definition** |
| --- | --- |
| Concepts | Concepts refer to how the treatment material is thought of and communicated to the facilitators, intervention participants, community members, and other stakeholders. The program’s and the facilitator’s credibility may be reduced if the communication of concepts and the concepts themselves do not match the local culture. |
| Methods | Methods are the procedures followed to achieve treatment goals. These methods and procedures should be congruent with the participants’ culture and use of language. |
| Goals | Goals are the agreement between participants and facilitator in what participants would like to achieve during the course of the treatment. These goals must be realistic and fit with the participants’ values and personal motivations. |
| Context | Context refers to the participants’ economic, social, political and cultural environment. This should look beyond just the participant as an individual and focus on outside factors, such as socialization, discrimination and family history, that could influence the treatment. |
| Content | The knowledge, values, customs, and traditions shared by the participants should be integrated into all elements of the treatment. This can be seen as a starting point for culturally adapting the recruitment process, assessments, and the treatment itself. |
| Metaphors | Culturally appropriate symbols or concepts should be embedded within the intervention that support participants in absorbing the treatment’s core mechanisms of action. Metaphors used may be pictorial, idioms, commonly used phrases or item and symbols. |
| Persons | A culturally appropriate intervention must consider the role of ethnicity, race, gender, class and other relevant social constructs in the relationship between the participants and facilitators. This relationship should respect expectations and limitations that are reflective of the local culture. |
| Language | Language is inherently attached to culture and is related to the expression of emotional experiences. The intervention should be in the language most comfortable and accessible to the participants and should also use appropriate terminology based on the education levels of the facilitators and participants. |

**TABLE 2.** Ecological Validity Model (EVM) Matrix for Adaptations

| **Adaptation Dimension** | **Pages** | **Implementation Strategy** | **Rationale for Change** | **Evidence for Change** | **Description of evidence base** | **Status of change** |
| --- | --- | --- | --- | --- | --- | --- |
| Language  People  Metaphors  Content  Concepts  Goals  Methods  Context | Note where in the intervention manual this change should occur | Description of what exactly should be changed in the manual, or other intervention material | Description of why it should be changed and what this change would accomplish for the intervention | Note what steps in the adaptation process provided evidence for this change (ex. 1 – Identifying mechanisms of action, 2 – literature review, 3 – ToT etc.) | Describe the evidence that suggested this change | Note the status of creating this change in the manual or other intervention material (ex. In-process, completed etc.) |

**FIGURE 1.** *Tension* Toolkit


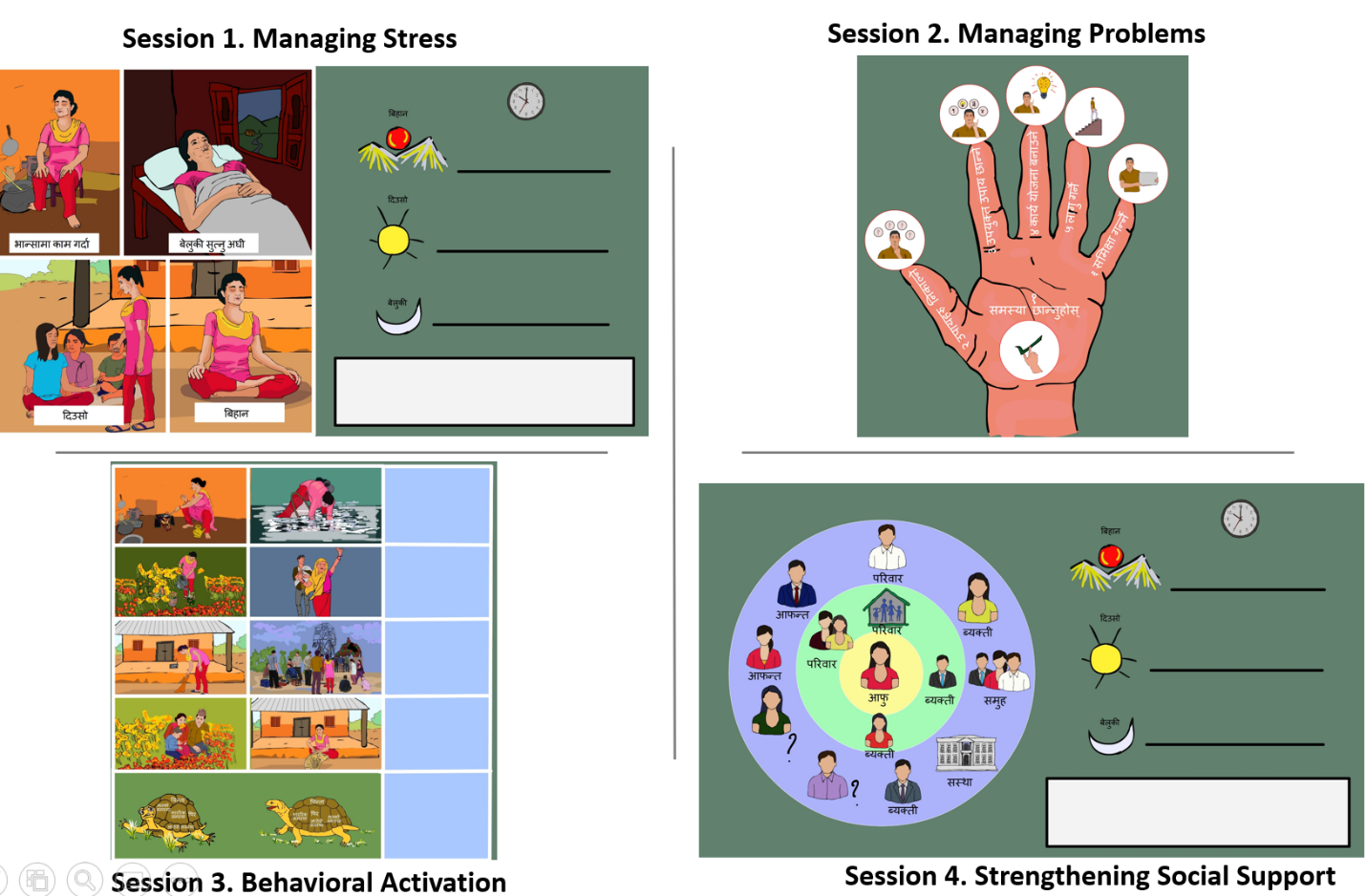


*Note: Session 1: Depicts female participant practicing deep breathing technique during different times of the day. On the right of these pictures, there is space for participant to note what time of day they plan to practice the technique and note the number of times they practiced the technique throughout the week. Session 2: Depicts 6-steps for the managing problems technique delivered in session s2. Session 3: Depicts female participant taking part in several pleasurable activities such as fishing, dancing, and spending time with family. The card also has a picture of the “tension turtle”; one who is unable to manage their heavy shell and the other who is walking slowly but steadily even while carrying a heavy load. Session 4: Depicts a “me map” of various people, organizations and other support systems that participants can identify for personal support.*
